# The Role of the Environmental Risk Factors in the Pathogenesis and Clinical Outcome of Atopic Dermatitis

**DOI:** 10.1155/2019/2450605

**Published:** 2019-04-21

**Authors:** Domenico Bonamonte, Angela Filoni, Michelangelo Vestita, Paolo Romita, Caterina Foti, Gianni Angelini

**Affiliations:** ^1^Section of Dermatology, Department of Biomedical Science and Human Oncology, University of Bari, 11 Piazza Giulio Cesare, Bari 70124, Italy; ^2^San Gallicano Dermatologic Institute, Via Elio Chianesi 53, 00144 Rome, Italy; ^3^Section of Plastic and Reconstructive Surgery, Department of Emergency and Organ Transplantation, University of Bari, 11 Piazza Giulio Cesare, Bari 70124, Italy

## Abstract

Atopic dermatitis (AD) prevalence is rising worldwide. Literature data suggest the incidence of AD in developing countries is gradually getting close to that of developed ones, in which AD affects 20% of the paediatric population. Such an increment, associated with significant variations in prevalence among the various countries, underlines the importance of environmental factors in the disease onset. Among these, great importance is given to hygiene, intestinal microbiota, exposure to bacterial endotoxins, outdoor living with contact to animals, atmospheric pollution, weather, and diet. Genetic (alteration of the skin barrier function) as well as immunologic factors concur with the environmental ones. Only the systematical study of all these elements can best elucidate AD epidemiology.

## 1. Introduction

Atopic dermatitis (AD, also atopic eczema) is the most common inflammatory disease in childhood and poses a great number of problems related to health and quality of life of patients [1–9]. An increase in the prevalence of AD is seen all over the world and accordingly causes much general interest in the identification of potential risks and protective environmental factors.

From 1990, the number of studies dedicated to the epidemiological research of AD has increased eightfold [7]. The data about its prevalence collected by the International Study of Asthma and Allergies in Childhood (ISAAC) of 2 million children in 106 nations (the most comprehensive study of global scope) support an increase in most developing countries, in particular among 6-7-year-olds [6, 10–13]. In particular, they have been highlighted in children estimates of 10-12% throughout the United States, up to 20% in some US states [14, 15], and 7-10% in the adult population [14, 16]. A recent systematic review of 69 studies also confirmed that AD is a “worldwide phenomenon” with a life prevalence of well over 20% and a significant increase in low-income countries of Africa and East Asia [17–21].

All these studies show that the well known differences in the prevalence of AD, not only between different nations but also within the same country, are gradually levelling out as a consequence of the globalisation process [22]. In fact, for example, after the reunification of the two Germanys, East Germany, within a short time span, saw an increase of AD from 16% in 1991 to 23.4 % in 1997 [23]. Similar observations associated with urbanization in developing countries and migration of populations from areas with low prevalence to areas with high prevalence of AD have emerged [24, 25].

Thus, analysis of these data seems to reasonably suggest an important role of environmental factors in the pathogenic mechanism of AD together with genetic and immunologic ones.

## 2. Environmental Factors

Table 1 shows the risk factors that influence AD, some of them with a preventive effect, others aggravating.

### 2.1. Climate

Climate, a factor that potentially could explain the differences in prevalence between different populations, has received scant attention in relation to AD. Data on association with prevalence AD and temperature are conflicting [9, 26–29].

From an ISAAC Phase One study, where the variables latitude, altitude, average outside temperature, and relative outside humidity were factored in, it became apparent that symptoms of AD correlate positively with latitude and negatively with annual outside temperature [30]. These results are confirmed by other similar studies in Spain [31], Taiwan [32], and the USA [33]. UV light has a well known immunosuppressive effect [34], in part related to the fact that it facilitates the conversion of trans-urocanic acid in the filaggrin (FLG) of the skin barrier into cis-urocanic acid, with immunosuppressive effect [7, 35]. Also, because exposure to the sun/UVB increases serum levels of vitamin D, it can logically be assumed that the clinical improvement of AD through sun exposure can be mediated at the molecular level by the same vitamin. This data point is supported by the observation that vitamin D deficiency is associated with the presence of more severe AD manifestations in skin areas not exposed to light, which demonstrates a protective local effect of vitamin D [36]. Low outside temperatures, especially in combination with skin irritants, are responsible for an aggravation of eczema [26]. It is also true, however, that in some cases an aggravation of the disease occurrs during the summer [37]. The climatic factors, therefore, need further study, also in relationship with pollen and skin barrier function.

### 2.2. Urban vs Rural Life

It is known that in populations of the same ethnicity and genetic background the risk of AD is higher in cities than in the countryside [38]. This contrast between city and country life is supported by the systematic review of 26 studies [39]. Environmental risk factors, which need to be considered relevant, are urbanisation, differences in hygiene, microbial infections, vaccination, use of antibiotics, environmental pollution, exposure to allergens, and diet. Further studies are needed in order to identify the risk factors of urban living in development of AD.

### 2.3. Diet

Given the fact that AD is still not very common in developing countries, we can ask if a “Western diet” (i.e., high intake of refined cereals, red and preserved meats, and saturated and unsaturated fatty acids) is a possible contributor to the increase in the disease. One ISAAC Phase Three study showed a significant protective effect of the frequent intake of fresh fruit (1-2x/week) and an aggravating effect of fast-food intake (≥ 3x/week) [40]. Another ISAAC study came to similar conclusions, highlighting an inverse association between the prevalence of AD and the* per capita* intake of vegetables, cereal proteins, and fresh and frozen fish [41]. This is confirmed in other studies, which show that a high intake of fish during pregnancy lowers the risk of AD in the first 5 years of life by 25-43% [42, 43]. A similar risk reduction was also reported in children with high intake of fish during late childhood [44, 45]. The protective effect of fish can be attributed to its high content in n-3 polyunsaturated fatty acids (n-3 PUFA), which is positively correlated with anti-inflammatory activity. The Western diet of the last decades is poor in n-3 PUFA, while proinflammatory n-6 PUFA, such as linoleic acid, is increased [46]. This hypothesis is corroborated by studies showing that maternal consumption of n-6 PUFA during pregnancy is associated with an increase of AD in Japanese children of 2 years of age, and the consumption of margarine rather than butter in children leads to an increase of AD [40, 46, 47].

Despite some conflicting results [48, 49], case-controlled studies of AD sufferers have shown higher blood levels of linoleic acid (precursor of n-6 PUFA) and lower levels of n-3 PUFA [50, 51]. However, from studies in literature concerning the effects of diet on AD [52–56] emerges that a strict diet management is not effective in general in the treatment of AD [57]. Further studies are therefore needed in this regard [9].

### 2.4. Breastfeeding and Weaning

It is a common belief that breastfeeding prevents allergies, including AD. The World Health Organization (WHO), in fact, recommends exclusive breastfeeding for the first 6 months and the European Ministries of Health advise the same, i.e., exclusive breastfeeding for at least 4 months, to prevent allergies [58, 59]. However, ISAAC Phase two studies conducted both in developed and in developing countries including 51.119 school-age children show only modest support for this thesis [60]. Systematic research on various populations also did not show a statistically significant benefit of exclusive breastfeeding [61–66]. Further studies are necessary to determine the role of breastfeeding in childhood AD and the relation between breastfeeding and introducing solid foods.

### 2.5. Obesity and Exercise

A growing number of children in affluent societies are overweight. Various research suggests both an association and a dissociation between obesity and AD [67–78]. Even time spent in front of television (≥5 hours) has a positive association with the risk of AD, the same being stronger in obese* vs* overweight* vs* underweight/normal children with respect to time of exposure and response [79]. It remains to be established whether these positive associations are causal, e.g., linked to inflammation of adipokines (molecules synthesised and secreted by adipose tissue), such as leptin and adiponectins, or related to dietary factors, that may encourage the development of AD through oxidative stress, since diets that exclude antioxidant foods, such as fruits and vegetables, are related to an increase in obesity and AD.

### 2.6. Air Pollution

Air pollution is the source of a wide variety of substances derived from industrial and nonindustrial processes. In normal conditions, air pollution usually excludes derivatives of natural phenomena such as volcanic eruptions and smoke of spontaneous forest fires, or radioactive material from military tests, and batteries. Mould and spores, however, are sometimes included because of the allergological damage they cause in a large population segment [80]. Air pollutants can originate from indoor and outdoor environments and can penetrate the skin, binding to the stratum corneum, entering the systemic circulation [81].

Since a large portion of AD cases, about 1/3, is observed in the first year of life, it is imperative to consider the impact of prenatal exposure to air pollution. In a rather complex study involving 469 pregnant women, prenatal exposure to fine particulate matter (PM 2.5) and consequently postnatal exposure to the same and to cigarette smoke were monitored every 3 months for 1 year [82]. It showed that the prevalence of AD during the first year of life doubles in presence of a high prenatal exposure to fine particulate matter and postnatal exposure to tobacco smoke. It also showed that high amounts of fish intake (>205g/week) rich in n-3 PUFA with anti-inflammatory action during pregnancy reduce the risk of AD by 25-43%. The same risk is further reduced when the intake of fish continues in the first years of life [83].

Another long-term study conducted with sufferers of AD in early childhood (from 3 months to 8 years) which took into account a variety of daily parameters (NO2, particulate matter, volatile organic compounds such as benzene, toluene, xylene, and styrene, temperature, and relative humidity), showed a significant SCORAD deterioration in the presence of high concentrations of particulate matter, toluene, and other volatile organic compounds. In particular, AD got worse in spring with high levels of styrene, in summer with high levels of toluene and NO2, in autumn with high levels of volatile organic compounds, and in winter with high levels of fine particulate matter [80]. These data were confirmed by other researches [84–88].

Confirming the “outdoors” observations above, a move “indoors” into a clean house (reduction of fine particulate matter from 182,7 to 73.4 *μ*g/ m^3^) and a hospital (in a “low pollutant room” after just 3-4 days) significantly improves the SCORAD of AD sufferers [89, 90]. A Swedish study showed a dose-dependent association between AD and lower ventilation in the houses, in particular, in the child's bedrooms [91]. A German study found association between indoor renovation activities (painting, floor covering, and new furniture) before birth and in the first years of life and lifetime prevalence of AD, likely in connection with high levels of volatile organic compounds (VOCs) [92]. Cleanliness that needs to take into consideration chemical, physical, and biotic (dust, mites, microorganisms, particulates, volatile organic compounds, temperature, and relative humidity) parameters should be observed not only in the home and in hospitals, but also in kindergartens and schools. From the aforementioned data, it is clear that outdoor and indoor pollution can trigger and/or exacerbate AD.

The biomechanism of the effect of particulate matter is not entirely clear. It contains a great variety of toxic substances (polycyclic aromatic hydrocarbons, tobacco smoke, alloy smoke, and organic compounds derived from traffic, sulfates, nitrates, and metals). In particular, fine particulate matter crosses through the placenta, the skin, and the respiratory tract, has a slow index of sedimentation (and therefore remains suspended in the air for a long time), and has a “carrier” effect for dust mites and pollen due to its ability to link to proteins. Prenatal exposure to polycyclic hydrocarbons has various negative effects, such as the production of free radicals, activation of apoptosis, and the production of IgE and Th2 cytokine. Postnatal exposure increases the effects of prenatal exposure and likely damages the skin barrier, with a resulting inflammatory process [82–84, 93–99].

### 2.7. Tobacco Smoke

A controlled case study (83 patients/142 control subjects) showed a direct relationship between the cumulative number of cigarettes and the onset and/or worsening of AD in adults [100]. The same significant relationship between environmental tobacco smoke and AD onset was also highlighted in nonsmokers. It is known that AD in adults more often takes on clinical character such as prurigo, involving especially the face and hands, and is associated with high values of IgE, asthma, and allergic rhinitis. From various studies it emerges that AD is significantly associated with active and passive smoking also in adolescents [101–106]. Under an immunological profile, tobacco smoke increases the levels of proinflammatory cytokines and reduces those of anti-inflammatory cytokines [100]; it causes oxidative damage, decreases skin barrier function [107, 108], and has an irritant effect on the skin [9].

### 2.8. Ozone

Measured daily for about 2 years from 10 am to 6 pm, an excess of ozone (formed by the action of UV rays on the oxygen of the air) aggravates various skin conditions, thus requiring a greater number of visits to the doctor. After 7 days of increased ozone levels there is a significant increase of 3,84% of visits for AD, of 2.86% for contact dermatitis, and of 0.8% for urticaria [109]. Ozone is a highly unstable and therefore very reactive oxidant; it reacts with the biomolecules of the skin and forms ozonides and free radicals. Low nontoxic doses of ozone increase the production of antioxidants, while high doses act as a proinflammatory cytokine [109, 110].

### 2.9. Skin Barrier and Allergic Sensitization

Recently, the strong association between genetic mutations of FLG in the epidermal barrier and atopy has attracted considerable interest with regard to the role, which alterations of this barrier might play in the development of AD and sensitisation [111–113]. The current hypothesis is that in subjects without skin barrier defects the epidermis is in a state of integrity with resulting normal “transepidermal water loss” (TEWL) and adequate protection from microorganisms and environmental allergens. Genetic mutations of FLG increase TEWL and are associated with sensitisation to aeroallergens and food [114–118]. In a similar context, the skin barrier acts as a mediator of sensitisation, which therefore becomes predominantly a “secondary phenomenon” in AD and a significant cause of aggravation and chronicity of the same.

### 2.10. Microbial Exposure

In two recent literature reviews, the relationship between microbial exposure and the risk of AD has been studied [119, 120]. The argument of the risk of the disease inversely related to hygiene has been widely studied in response to observations in different scenarios since the end of the 1980s [9, 121–124].

#### 2.10.1. Hygiene

Many studies concerning the risk factors in AD regard the “hygiene hypothesis.” One study conducted on a very large cohort (> 10,000) of infants, which took into account the level of hygiene at 15 months (frequency of washing, use of household detergents, and baby wipes), showed a proportional increase in the risk of diseases between the age of 2.5 and 3.5 years with an increased level of hygiene [125]. One Japanese study, on the contrary, on a cohort of 865 subjects, has found an inverse relationship between daily baths or showers* vs* less frequent ones [126].

#### 2.10.2. Daycare Centers

The stay in nurseries seems to be associated with increased microbial exposure, in particular with respiratory infections, and some authors have reported a reduction in the risk of AD in children attending daycare in the first year of life [127, 128]. Others, however, have found the opposite effect [129, 130].

#### 2.10.3. Animals and Farm Life

Various studies conducted in this area did not show a convincing protective effect of this lifestyle [131–137]. It is a very interesting fact that the consumption of unpasteurized farm milk during the first 2 years of life is an independent protective factor against the development of AD, even in families not living in the countryside. This inverse relationship is also independent from a family history of allergies. With boiling, cow's milk loses its protective effect [134, 138, 139]. The mechanism of this protective effect remains uncertain and could be related to microbial contamination or other constituents of nonprocessed milk [139–141]. It has also been shown that direct contact with farm animals reduces the risk of AD in the first years of life, in particular where the mothers have regular contact with farm animals during pregnancy; this protective effect appears even more pronounced in those exposed during their prenatal life rather than postnatally [135, 136, 142], confirming that innate immunity may have a particular importance. This is causal for the well known argument about the contrast between “country child” compared to “city child.”

#### 2.10.4. Pets

Many studies have been conducted on this subject [143–147]. They all found dogs to have a protective effect, especially in the first years of life [147, 148]. The role of cats is not as clear: where the mutations of FLG of the skin barrier are considered, there is a significantly higher risk of AD in those with mutations compared to children without mutations, suggesting that cat sensitisation may be favoured by a compromised skin barrier, which in turn contributes to the risk of AD [145, 146, 149].

#### 2.10.5. Bacterial Endotoxins

Risk reduction through the exposure to farm animals and dogs, in particular during pregnancy, is attributed to endotoxins (lipopolysaccharides on the surface of Gram-negative bacteria), also because they are known to induce IL-10 and INF-gamma [150]. Cohort studies have shown a risk reduction of AD of up to 50% with exposure to bacterial endotoxins [151–153], with the effect limited to high levels of exposure and/or the first year of life [151, 152].

#### 2.10.6. Helminthes

The protective effect of helminth infections (*Ascaris lumbricoides*) on AD risk was demonstrated in a double-blind randomised controlled study with antiparasitic therapy conducted on more than 2500 pregnant women in an endemic helminth area in Uganda (these situations are today endemic in tropical areas due to lack of hygiene) during the last trimester of pregnancy: the AD risk up to the first year of life is increased by about 2 times in the treated group [154]. It was also observed that lack of exposure to helminths seems to have no effect in subsequent years of life, confirming that the innate immune system protects from the risk of AD [155, 156].

#### 2.10.7. Paediatric Infections and Vaccinations

A recent analysis of respiratory viral infections showed an AD risk reduction associated with prenatal exposure to the same infections, in particular in the last trimester of pregnancy and in the first 7 months of postnatal life [157]. Other studies, however, have not confirmed the same results [158, 159]. As far as viral and bacterial childhood infections (chicken pox, mumps, measles, and whooping cough) are concerned, the majority of studies show positive observations or no correlation [160–165].

#### 2.10.8. Antibiotics

The antibiotics used in respiratory, gastrointestinal, and aural infections, rather than the infections themselves, are causally responsible for the risk of AD development [166]. The same risk is increased by 41% in those receiving at least one cycle of antibiotics in the first years of life [167]. There is also a significant dose-related association with an increased risk of 7% for each additional cycle of antibiotics with a particularly strong effect of broad-spectrum antibiotics [168]. It is possible that the increase in risk from antibiotics is due to microbiome alterations in the host, with a subsequent alteration of the immune system or an increased immunological response to environmental allergens.

#### 2.10.9. Microbiome of the Intestinal Tract and the Skin

The intestinal microflora of children in the first years of life that subsequently develop AD has mostly* Staphylococcus aureus* and coliform bacteria and less lactobacillus and bifidobacteria [169, 170]. After intrauterine sterility, skin, intestines, and respiratory tract are colonised immediately after birth by a broad spectrum of bacterial agents [171]. Various studies analyze the effect of the gut microbiota on the onset and severity of AD. However, the results of these studies are conflicting: some are in favor of a positive effect of probiotics on the severity of AD, with concomitant alteration in the gut microbial composition; others show no effects of probiotics on the severity of AD despite a concomitant change in the gut microbial composition [172–178]. According to some studies, the subjects with AD have a different gut microbiome compared to healthy individuals, while according to others there would be no differences. Various reasons can explain these conflicting results: methodological differences, difficulties in isolation and identification of gut bacterial species, and complexity of interaction between the gut microbiota and external factors [178]. Therefore the role of the gut microbiome in AD remains rather controversial; further studies are needed in this regard [178].

## 3. Interaction between Environmental Factors, Genetic Factors, and Immune System

To better understand the various problems so far exposed, it is important to study the relationship between environmental factors, genetic factors, and immunological factors. In addition to those genetic factors involving FLG, there must be an involvement of other genes, since more than 50% of subjects with AD do not show FLG mutations [179, 180]. Another example of genetic-environmental interaction is provided by the observation that exposure to endotoxins reduces the risk of AD and sensitisation only in subjects with a specific phenotype of the lipopolysaccharide receptor CD14 encoded in chromosome 5q31.1 [153]. Along the same line, other studies on the impact of environmental exposure to farm life in the first years of life showed a strong innate immunity response through the regulation of the expression of CD14, TLR2, TLR4, TLR5, and TLR9 receptors, not only at the level of peripheral blood cells, but also in leukocytes in the blood of the umbilical cord, with dose-related effect in response to exposure to a high number of farm animals and consumption of unpasteurized milk [139, 142, 181]. These results are confirmed in the observation that maternal consumption of unpasteurized cow's milk modulates the production of cytokines in young age children [141].

## 4. Conclusions

The epidemiology of AD has made considerable progress in recent years with studies on a global scale. Surely, the more the conducted studies are, the more complex this disease appears: we expect them to have to do with several distinct entities with the same clinical manifestations rather than with a single disease. The increasing global prevalence of AD cannot be attributed to genetics alone, suggesting that environmental factors may trigger or flare dermatitis in predisposed subjects. On the other hand, it is doubtful that the environment exposure that is harmful in AD may be sufficient to cause the affliction without the underlying predisposition. Rather, environmental risk factors have pruritogen and inflammatory action, besides worsening skin barrier function. The future research, therefore, should be addressed to study in depth the gene-environmental interaction in order to better understand the complex pathophysiology of AD.

Understanding the mechanisms of the environmental risk factors is crucial for the therapeutic targets and the prevention of the disease. For example, recognition of the role of microbiome or exposure to climate, food, and other exogenous factors may result, respectively, in new treatment approaches [182] and new treatment strategies to prevent flares [183]. Other main areas for future research are furthermore the protective effect of the unprocessed cow's milk and helminth parasites [7].

In conclusion, the pathophysiology of AD seems more complex than up to now recognized. It is mandatory over the next future to define the role of the various inflammatory pathways, as well as the impact of environmental risk factor on cutaneous inflammation in AD. New methods for assessing the genotypes and clinical phenotypes of AD will allow to identify the various patient subsets with regard to the various protective and aggravating environmental factors which enter into the determinism of the disease [9, 184].

## Figures and Tables

**Table 1:** Protective and aggravating factors in the determinism of atopic dermatitis.

*(1) Environmental factors*

Climate (temperature, UV, humidity, and precipitation)
Urban vs rural life
Diet
Breastfeeding and weaning
Obesity
Physical exercise
Atmospheric pollution (outdoor, indoor pollutants)
Tobacco smoke
Ozone

*(2) Cutaneous barrier and allergic sensitization *

*(3) Microbial exposure *

Hygiene
Animals and farm life
Pets (dogs)
Bacterial endotoxins
Helminthes
Paediatric infections and vaccinations
Antibiotic exposure
Microbiome of the intestinal tract and the skin
Probiotics and prebiotics
